# Thermosensitive Behavior and Super-Antibacterial Properties of Cotton Fabrics Modified with a Sercin-NIPAAm-AgNPs Interpenetrating Polymer Network Hydrogel

**DOI:** 10.3390/polym10080818

**Published:** 2018-07-25

**Authors:** Yifan Cui, Zijing Xing, Jun Yan, Yanhua Lu, Xiaoqing Xiong, Laijiu Zheng

**Affiliations:** 1 Department of Textile and Material Engineering, Dalian Polytechnic University, Dalian 116034, China; Evan_C94@163.com (Y.C.); m18742054960@163.com (Z.X.); xxq890108@163.com (X.X.); fztrxw@dlpu.edu.cn (L.Z.); 2 Key Laboratory of Functional Textile Materials, Eastern Liaoning University, Dandong 118003, China

**Keywords:** sericin hydrogel, nano-silver, antibacterial activity, thermosensitive, cotton fabrics

## Abstract

Poly(*N*-isopropylacrylamide) (PNIPAAm), sericin (SS), and silver nitrate were combined to prepare an interpenetrating network (IPN) hydrogel having dual functions of temperature sensitivity and antibacterial properties. The structure and size of AgNPs in such an IPN hydrogel were characterized by the Fourier Transform Infrared spectrum (FT-IR), X-ray powder diffraction (XRD) and Transmission Electron Microscope (TEM), and the thermal properties of the IPN hydrogel were characterized by Differential Scanning Calorimetry (DSC). Based on XRD patterns, Ag^+^ was successfully reduced to Ag^0^ by SS. It was observed by TEM that the particle size of silver particles was lower than 100 nm. The glass transition temperature (T_g_) of IPN hydrogel was better than that of the PNIPAAm/AgNPs hydrogels, and lower critical solution temperature (LCST) values of the IPN hydrogel were obtained by DSC i.e. 31 °C. The thermal stability of the IPN hydrogel was successfully determined by the TGA. This IPN hydrogel was then used to modify the cotton fabrics by the “impregnation” method using glutaraldehyde (GA) as the cross-linking agent. The structures and properties of IPN hydrogel modified cotton fabric were characterized by scanning electron microscopy (SEM), FT-IR, and the thermogravimetry analysis (TGA). The results show that NIPAAm was successfully polymerized into PNIPAAm, and that there were neglected new groups in the hydrogel IPN. The IPN hydrogel was then successfully grafted onto cotton fabrics. SEM observations showed that the IPN hydrogel formed a membrane structure between the fibers, and improved the compactness of the fibers. At the temperature close to LCST (≈31 °C), the entire system was easily able to absorb water molecules. However, the hydrophilicity tended to decrease when the temperature was higher or lower than the LCST. The antibacterial rates of the modified cotton fabric against *S. aureus* and *E. coli* were as high as 99%.

## 1. Introduction

Cotton fabrics are common natural textiles which are widely used for their low price, hygroscopicity, gas permeability, and softness. However, these characteristics provide a favorable environment for bacteria, facilitating their growth and reproduction on cotton fabrics [1]. Thus, the demand for antimicrobial properties of textiles is increasing. Antibacterial materials play a key role in antibacterial agents, which inhibit the growth and reproduction of bacteria or even kill them [2].

Generally, antibacterial agents are divided into three major categories: natural, organic, and inorganic. Among them, inorganic antibacterial agents have become the focus of research into antibacterial agents because of their high safety, heat resistance, and durability [3]. Silver is a highly effective inorganic antibacterial agent having low toxicity and wide sterilization, etc. [4]. Its antibacterial effect is greater than other inorganic antibacterial materials such as Cu, Hg, and Zn [5,6,7]. However, in nature, silver exists in a free state; it is also stable in air and water. Consequently, silver ions that cannot exhibit antibacterial properties are difficult to reduce to silver. Therefore, the choice of reduction method from among the chemical, physical, and biochemical methods, becomes important [8,9]. Chemical reduction is the most commonly-used method for preparing silver nanoparticles (AgNPs). Its principle is to react free silver ions with appropriate reducing agents such as zinc powder, hydrazine hydrate, sodium citrate, and sericin (SS) in the liquid phase to reduce them to silver. However, silver impurities at a nanoscale prepared by this method are relatively high in content and agglomerate easily. Therefore, there is a need to add dispersants to reduce the agglomeration of AgNPs. In recent years, nano silver has found many novel and facile synthesis methods. Ag nanoparticles have been immobilized onto hollow fiber membranes which are used to improve antibacterial properties [10]. Ag nanoparticles have also been used in combination with carboxybetaine functional composite membranes to endow them antibacterial and antifouling properties [11].

Presently, green chemistry has become an important topic. The key to the preparation of metal nanoparticles lies in the selection of environmentally friendly reducing agents and non-toxic nanoparticles [12,13]. Raveendran et al. used soluble starch as a template and β-d-glucose as a reducing agent to synthesize AgNPs in water [14]. Yue X et al. prepared AgNPs using SS as a reducing agent [1]. SS is one of the pollutants in silk processing process [15,16], and large amounts of SS emissions cause great pollution [17]. Compared with silk, SS has poor mechanical properties but a high degradation rate. For the sustainable development of resources, SS was used as the reducing agent and the dispersant in this experiment. There are many methods to carry silver on the surface of cotton fabrics, such as surface adsorption and carrier attachment. Desislava Staneva et al. synthesized a new type of composite cotton fabric by synthesizing a hydrogel containing AgNPs in two steps, and simultaneously synthesized AgNPs in situ under visible light irradiation [18]. BX Wang et al. used a chitosan hydrogel to modify the antibacterial properties of cotton fabrics [19].

Hydrogels as a three-dimensional polymeric network can absorb large amounts of water or biological fluids [20]. Therefore, hydrogels can absorb sericin solutions to reduce AgNPs, thereby acting as a blocker and preventing aggregation among AgNPs. Stimuli-responsive hydrogels are also a hot topics in recent decades; they can respond to physical or chemical stimuli, such as temperature, pH, and pressure [21]. Poly(*N*-isopropylacrylamide) (PNIPAAm) is currently the most widely-studied thermal-sensitive hydrogel. Its LCST is about 30–32 °C [22,23]. However, the grafting rate of pristine PNIPAAm hydrogel on cotton fabrics is poor [19]. Therefore, the addition of SS-containing active groups allows PNIPAAm hydrogel chemically to be grated with hydroxyl groups onto the fabric surface [24,25]. The addition of SS not only increases the grafting rate but also improves the hygroscopicity of natural fibers and makes them softer. 

As a common method for preparing hydrogels, Interpenetrating Polymer Networks (IPNs) technology was used in this article. It consists of two or more polymer networks that are physically entangled with each other [26]. PNIPAAm as a temperature-sensitive monomer was combined with SS and silver nitrate via radical polymerization to prepare a temperature-sensitive IPN. Then, the IPN hydrogel was used to graft onto cotton surface. The structure properties of the IPN hydrogel and the modified cotton were characterized. The grafting rate, temperature sensitivity, and antibacterial performance of the modified cotton were studied. This experiment is intended to prepare for the preparation of sustained-release antibacterial medical dressings in the future. The addition of a temperature-sensitive material can control the sustained release of nano-silver to achieve controlled release of nano-silver.

## 2. Experimental Section

### 2.1. Materials

*N*-isopropylacrylamide (NIPAAm, Energy Chemical, Shanghai, China, 98%), *N*,*N*-methylenebisacrylamide (BIS, Tianjin Aoran Fine Chemical Research Insititute, Tianjin, China, *M*_w_ = 154.17), Glutaric dialdehyde (GA, 50%, Damao Chemical Reagent Factory, Tianjin, China), Ammonium persulfate (APS, Damao Chemical Reagent Factory, Tianjin, China), *N*,*N*,*N*′,*N*′-tetramethylethylenediamine (TEMED, Sinopharm Chemical Reagent Co., Ltd., Shanghai, China), Silk sericin powder (SS, average molecular weight of 300~350, Hebei Senlangbio Technology Co., Ltd., Shijiazhuang, China), silver nitrate (AgNO_3_, 99.8%, Tianjin Day Feeling Chemical Technology Development Co., Ltd., Tianjin, China), Absolute ethanol (Damao Chemical Reagent Factory, Tianjin, China), Sodium carbonate anhydrous (Tianjin Ruijinte Chemicals Co., Ltd., Tianjin, China).

### 2.2. Synthesis of the IPN Hydrogel

**Figure 1:**
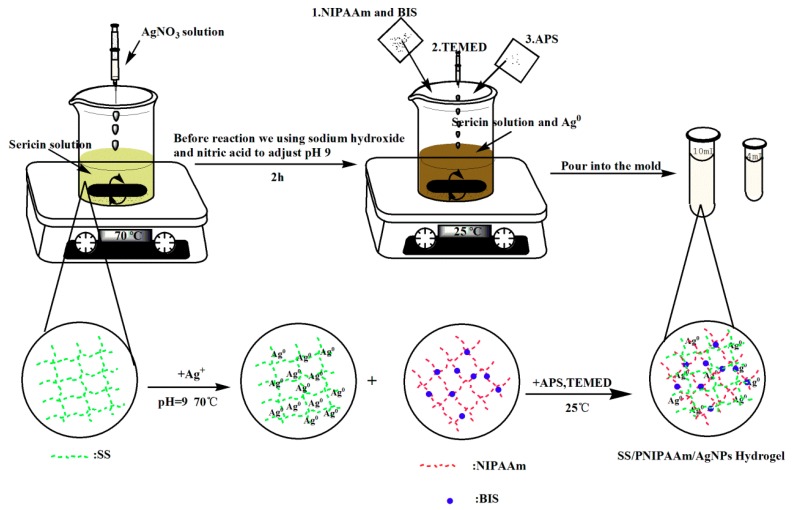
Synthesis scheme of the IPN hydrogel.

SS (0.5 g ± 0.005) was dissolved in 10 mL of deionized water and 1 mL of AgNO_3_ solution with a concentration of 50 mmol/L was added. The pH of the above solution was adjusted to 11 with a 1 mol/L of NaOH solution and HNO_3_ at 70 °C, and the reaction was terminated after the solution changed from light yellow to tan. After reducing the temperature to 25 °C, NIPAAm (0.5 ± 0.005 g) and BIS (0.02 ± 0.0005 g) were added and the solution was mixed thoroughly. Into the system, the initiator TEMED (6 µL) and co-initiator APS (0.005 ± 0.001 g) were added, mixed uniformly, and poured into the mold. The synthesis of IPN hydrogel is shown in Figure 1.

### 2.3. Grafting of the IPN Hydrogel onto Cotton

**Figure 2:**
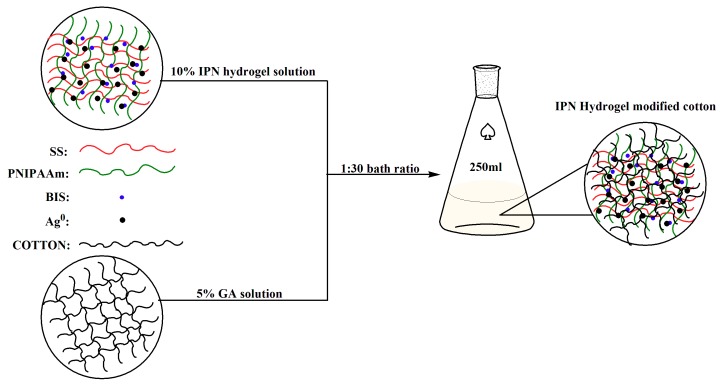
IPN hydrogel modified cotton process.

First, the cotton was pretreated (a piece of cotton was washed three times with ethanol and deionized water) to remove impurities and organic matter from the surface. Then, the pretreated cloth was boiled in a 10% aqueous solution of sodium carbonate for 2 h, washed with deionized water, and dried for later use. Next, the treated cotton was fully immersed in a 5% GA solution and further immersed in a 10% IPN hydrogel solution using an “impregnation” method at a bath ratio of 1:30 to graft modification of cotton. Finally, the modified cotton was vacuum dried for 5 min at 60 °C, vacuum dried 3 min at 150 °C, and dried at 60 °C to constant weight. The IPN hydrogel modified cotton process was shown in Figure 2.

### 2.4. Analytical Methods

The FT-IR spectra were measured using the Perkin-Elmer Spectrum 10 FT-IR spectrophtpmeter (Waltham, MA, USA) at room temperature from 4000 to 500 cm^−1^. Thermogravimetric analysis (TGA) was performed on a Linseis TGA/DSC (TA, New Castle, DE, USA). The surface morphology of cotton fabric samples were characterized by SEM (JSM-7800F, JEOL, Tokyo, Japan). The contact angle of cotton fabric was measured by K100C KRUSS Gmbh (Hamburg, Germany). The X-ray powder diffraction (XRD, XRD-7000S Shimadzu, Kyoto, Japan), Transmission Electron Microscope (TEM, JEM-2100 UHR, JEOL, Tokyo, Japan) and Dynamic Light Scattering (DLS, Malvern Zetasizer 300HSA, Malvern, UK) were used to characterize the structure and size of AgNPs in the IPN hydrogel.

### 2.5. Measurement of IPN Hydrogels Thermosensitive Behavior and LCST

The transmittance at 500 nm was measured using UV–Vis spectroscopy (UV-8000, Shanghai, China). The swelling IPN hydrogel was placed in a 10 mm × 10 mm × 40 mm cuvette. The temperature was raised from 25 to 60 °C, and every test sample was stabilized for 5 min before analysis at that temperature. 

All IPN hydrogel samples were immersed in distilled water at room temperature and swollen to Differential Scanning Calorimetry (DSC-Q2000, TA, New Castle, DE, USA). About 10 mg of the sample was placed in the DSC-Q2000; the heating rate was 2 °C/min, the temperature range was −20 °C to 60 °C, the test sample was kept under a nitrogen atmosphere LCST. About 10 mg of the sample was placed in the DSC-Q2000 at a heating rate of 20 °C/min, rising from 50 °C to 200 °C, naturally cooling to 50 °C, eliminating the heat history; then, the glass transition temperature (T_g_) of the sample was tested under a nitrogen atmosphere at a temperature increase rate of 10 °C/min from 50 °C to 200 °C.

### 2.6. Grafting Degree of Cotton Fabrics

The grafting rate of the modified cotton was calculated according to the following formula [19]:

(1) $$D_{G}\;\left(\%\right)=\frac{W_{\mathrm{aG}}-W_{\mathrm{aS}}}{W_{\mathrm{aS}}}\times 100\%$$

where D_G_ is the grafting rate, and W_aS_ and W_aG_ are the mass before graft modification and the mass after graft modification, respectively.

### 2.7. Measurement of Antimicrobial Activity of IPN Hydrogel Modified Cotton Fabrics

Calculation of live bacteria concentration: based on the number of colonies obtained from the two plates, the viable concentration in each sample flask was calculated according to Formula (2) (two effective numbers were retained).

K = Z × R(2)

where K is the concentration of viable bacteria in each sample flask (CFU/mL); Z is the average of the number of colonies of two plates; and R is dilution factor.

Modified Cotton Fiber Antibacterial Test: in this experiment, *E. coli* (ATCC 8099) and *S. aureus* (ATCC 6538) were selected as test strains. The antibacterial properties of IPN hydrogel modified cotton were quantitatively tested using the shake flask method (according to GB/T 20944.2-2007 and ISO/DIS 20743:2005 standards). Unmodified and IPN hydrogel-modified cotton were incubated at 37 °C for 24 h, and the percentage reduction of colony units was calculated by the following formula [27]:

(3) $$R=\frac{B-A}{B}\times 100\%$$

where R is the percentage of colony unit reduction, B is the number of bacterial colonies in the unmodified cotton (CFU/mL), and A is the number of bacterial colonies in the IPN hydrogel modified cotton (CFU/mL).

## 3. Results and Discussion

### 3.1. FT-IR Characterization of IPN Hydrogels

**Figure 3:**
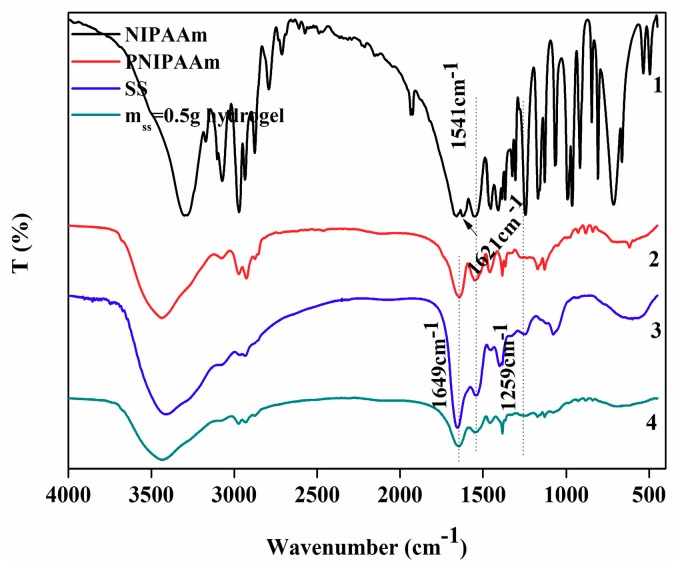
The FT-IR of (1) NIPAAm; (2) PNIPAAm; (3) SS; (4) m_ss_ = 0.5 g hydrogel. Note: in the previous experiments, an IPN hydrogel having a sericin quality of 0.5 g (hereafter referred to as m_ss_ = 0.5 g hydrogel) had good properties such as mechanical properties, thermal stability, etc. Therefore, m_ss_ = 0.5 g hydrogel was selected as the sample for characterization.

The FT-IR was used to determine the chemical structures of NIPAAm, PNIPAAm, SS, and IPN hydrogel. As Figure 3 shows, NIPAAm, PNIPAAm, and IPN hydrogel all had strong absorption peaks at 1649 cm^−1^, which is assigned to the characteristic absorption peak of C=O in Amide I band of NIPAAm and of carbonyl group in SS. The peak at 1541 cm^−1^ is attributed to N–H bending vibration and C–N stretching vibration in amide II bands of NIPAAm and the characteristic absorption peak of the amino group in SS. The absorption peak of NIPAAm at 1621 cm^−1^ was negligibly observed in any spectra, indicating that NIPAAm monomers were successfully polymerized into PNIPAAm [19]. By comparison, in the FT-IR spectra of the four substances, no new functional groups were added, nor did any disappear. Thus, the introduction of SS and silver nitrate did not affect the structure of PNIPAAm. SS, silver nitrate, and PNIPAAm were only physically connected to each other in the IPN hydrogel without chemical bonding.

### 3.2. XRD Characterization of IPN Hydrogel

**Figure 4:**
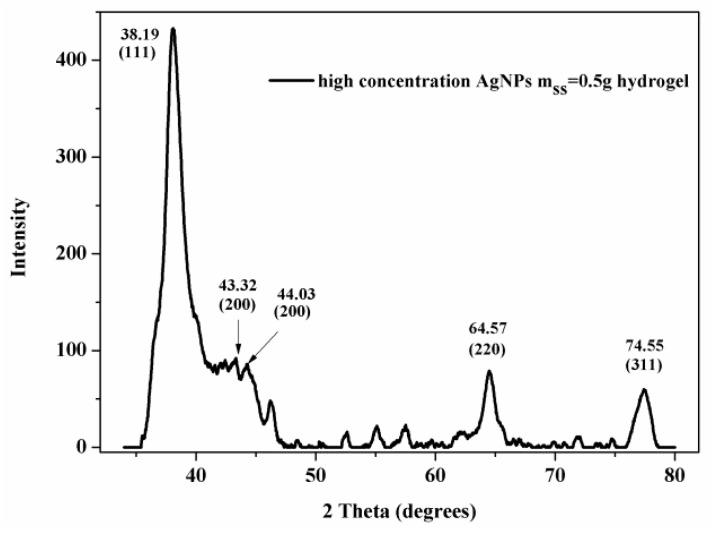
XRD of m_ss_ = 0.5 g hydrogel.

Figure 4 shows the XRD pattern of AgNPs. In the range of 20° to 80°, three strong peaks (38.39°, 64.57°, 74.55°), and a small number of peaks (43.32°, 44.03°) were shown, according to silver crystals 111, 200, 220, and 311. The results demonstrate that Ag^+^ was reduced to Ag^0^ by SS.

### 3.3. TEM and Dynamic Light Scattering Characterization of IPN Hydrogel

**Figure 5:**
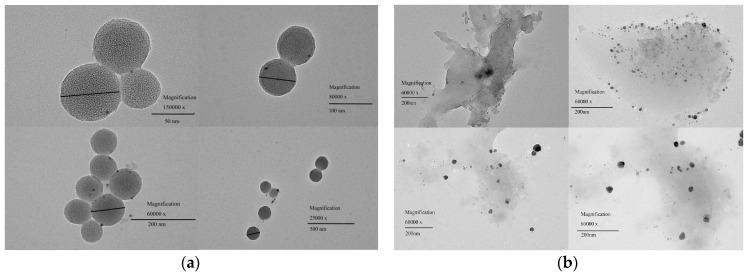
TEM of AgNPs and m_ss_ = 0.5 g hydrogel.

**Figure 6:**
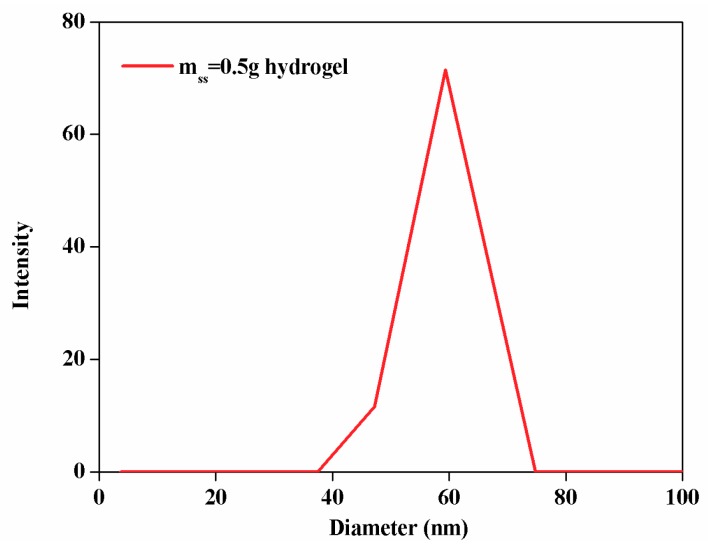
Dynamic Light Scattering of m_ss_ = 0.5 g hydrogel.

The morphology of AgNPs was characterized by transmission electron microscopy (TEM). Figure 5 shows TEM images of AgNPs (Figure 5a) and the hydrogel (Figure 5b). The average size of AgNPs was about 80 nm. By decreasing the particle size of AgNPs, their specific surface area is increased, thereby increasing the area in contact with bacteria. The later study focused on exploring the effect of this experimental process on the particle size of AgNPs. The particle size of AgNPs’ distribution diagram is shown in Figure 6. Obtained by Dynamic Light Scattering the AgNPs size is evenly distributed around 60 nm. The result is similar to the TEM result.

### 3.4. T_g_ Characterization of IPN Hydrogel

**Figure 7:**
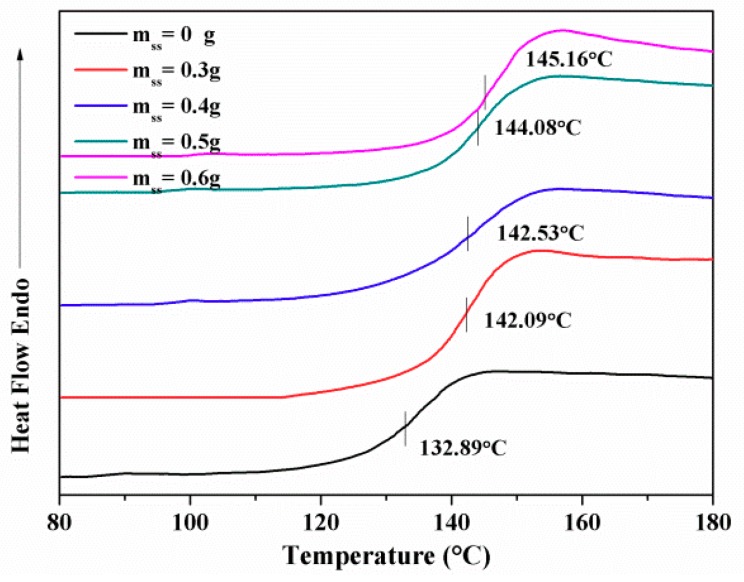
T_g_ of IPN hydrogels.

To investigate the effect of SS content on the thermal properties of the hydrogel, DSC was used to measure the T_g_. As Figure 7 shows, a single T_g_ was observed, because both components were compatible. The T_g_ of the IPN hydrogel increased from 142 to 145 °C with increasing SS content. When an interaction between SS and PNIPAAm occurs, a high temperature is required to promote the movement of macromolecular segments.

### 3.5. Thermosensitive Behavior of the IPN Hydrogels

#### 3.5.1. Transmittance Measurement of the IPN Hydrogels

**Figure 8:**
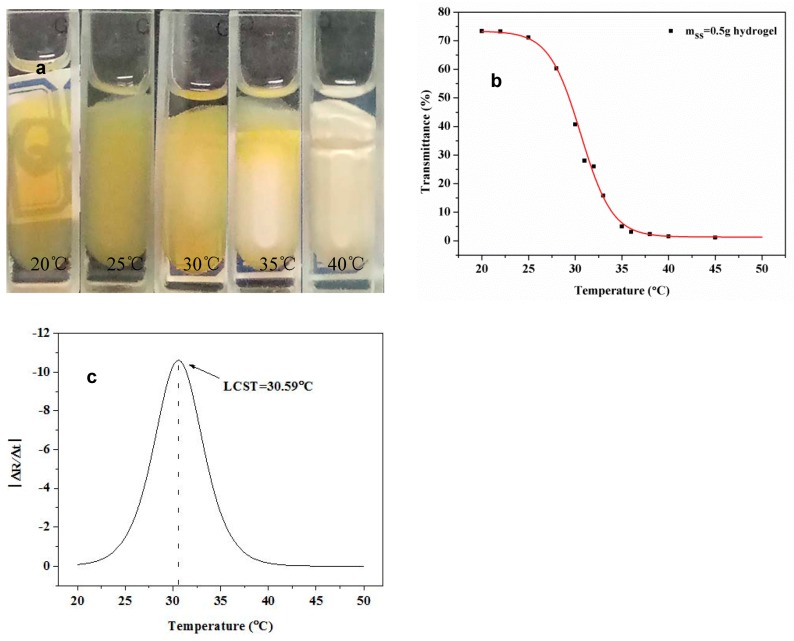
Photograph of m_ss_ = 0.5 g hydrogel at different temperatures (**a**), transmittance curve (**b**) and optical transmittance-temperature differential curve (**c**).

The change in the phase states of hydrogels at different temperatures can be represented by light transmittance. As Figure 8a shows, the color of the IPN hydrogel began to change at around 30 °C. By increasing the temperature, the IPN hydrogel gradually changed from a light yellow transparent state to a white opaque state, indicating greatly reduced transmittance. Therefore, the temperature sensitivity of the IPN hydrogel can be characterized by measuring the light transmittance of at different temperatures. As Figure 8b shows, the transmissivity of the hydrogel gradually decreased as the temperature increased. The highest point in the temperature-transmittance differential curve, as shown in Figure 8c, is the LCST. From this, it can be determined that the LCST of the IPN hydrogel having m_ss_ = 0.5 g was 31 °C, which agrees well with the DSC data. The LCST temperatures of the IPN hydrogel were all around 30 °C. 

#### 3.5.2. Determination of IPN Hydrogels’ LCST 

**Figure 9:**
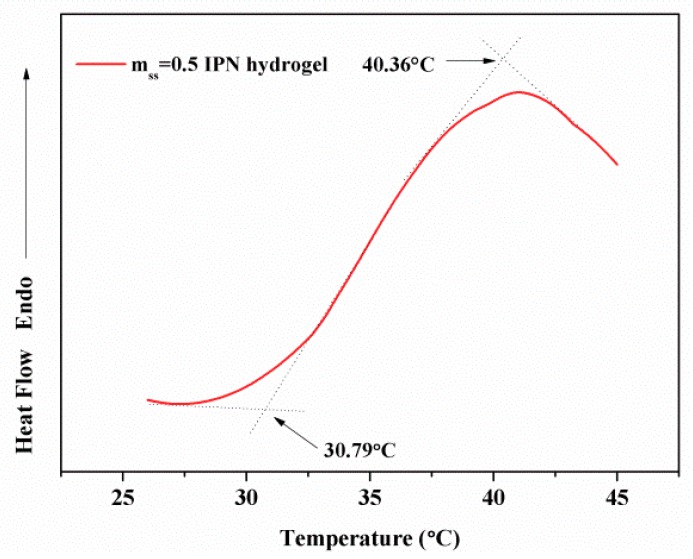
LCST of m_ss_ = 0.5 g hydrogel.

DSC was also used to measure the LCST of the IPN hydrogel. The DSC curve of the IPN hydrogel having m_ss_ = 0.5 g is shown in Figure 9. The intersection point between the onset temperature of the DSC curve is the LCST. The tangent on both sides of the peak is the maximum phase transition temperature. From Figure 8, the LCST value was 31 °C and the maximum phase transition temperature was 40 °C. This result was similar to the previous measurement (Figure 8).

### 3.6. Grafting IPN Hydrogel onto Cotton Fabric

#### 3.6.1. Analysis of the Weight Loss of the IPN Hydrogel Modified Cotton

**Figure 10:**
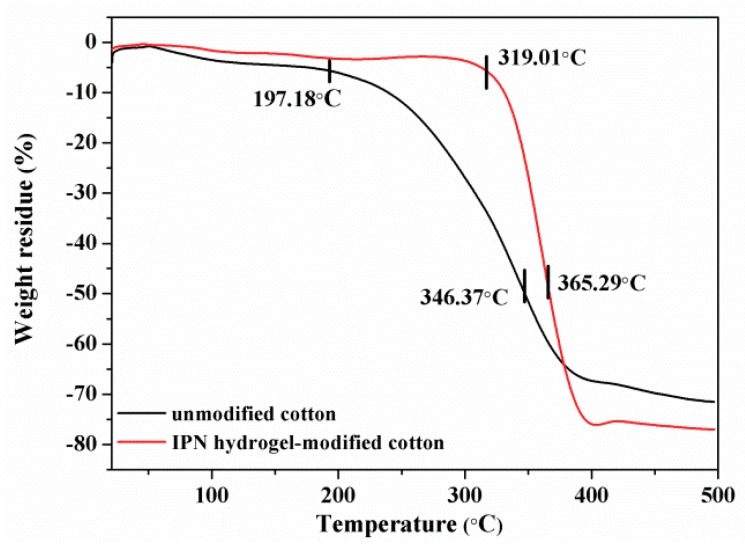
The TGA of cotton and m_ss_ = 0.5 gL hydrogel modified cotton.

TGA was used to characterize the thermal properties of IPN hydrogel modified and unmodified cotton (Figure 10). The temperatures at which the system loses 5% or 50% of its weight are set at the thermal decomposition starting temperature and semi-decomposition temperature. As Figure 9 shows, the thermal stability of the modified cotton was greatly higher than that of the unmodified cotton. The thermal decomposition starting temperature and the semi-decomposition temperature of the modified cotton were 319 °C and 365 °C, respectively. The unmodified cotton began to decompose at 197 °C, and reached the half decomposition temperature at 346 °C. Thus, the modified cotton presented higher thermal stability than did the normal cotton. The prepared IPN hydrogel can improve the structural compactness of the cotton. 

#### 3.6.2. FT-IR Characterization of IPN Hydrogel Modified Cotton

**Figure 11:**
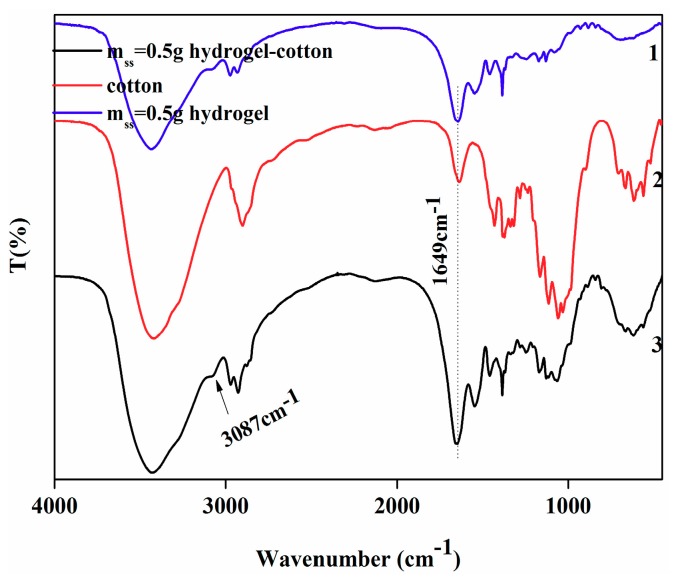
The FT-IR of (1) m_ss_ = 0.5 g hydrogel (2) cotton (3) m_ss_ = 0.5 g hydrogel cotton.

FT-IR was used again to determine the connection between IPN hydrogel and cotton. From Figure 11, the peak at 1649 cm^−1^ was the strongest in the IPN hydrogel modified cotton fabric because of the –COOH on the fabric and the –CHO on GA. The reaction produced a characteristic absorption peak assigned to a C=O group. The modified cotton added a characteristic peak at 3087 cm^−1^ The CHO reaction produced a characteristic vibrational absorption peak of the C=C group. This shows that the two aldehydes on GA can react with hydroxyl and carboxyl groups of SS in –OH and IPN hydrogels on cotton. Therefore, GA acted as a bridge between cotton and IPN hydrogels. Under the crosslinking of GA, IPN hydrogel can be grafted onto cotton surface. Therefore, after the modification of the IPN hydrogel, the cotton can develop temperature-sensitive properties.

#### 3.6.3. SEM Analysis of IPN Hydrogel Modified Cotton

**Figure 12:**
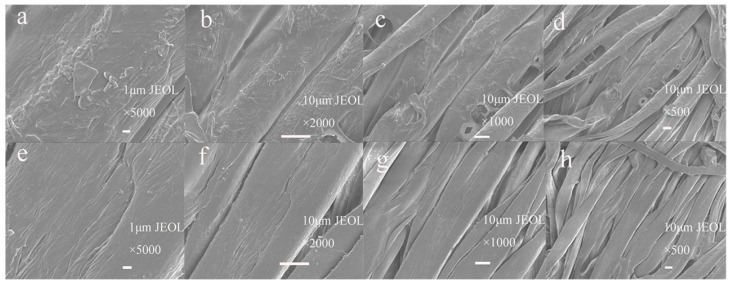
SEM of modified cotton (**a**–**d**) and unmodified cotton (**e**–**h**).

SEM and EDS were used to characterize the variations in the surface and elements content in the IPN hydrogel modified cotton and the unmodified cotton. A neglected link occurred among the fibers of the unmodified cotton (Figure 12e–h). The surface of the cotton modified by the IPN hydrogel formed a thin film covering the clear fiber interstices. The IPN hydrogel was evenly attached onto the cotton surface, causing neglected damage (Figure 12a–d). This result shows that the IPN hydrogel had good binding to the cotton. Table 1 shows the expected strong carbon and oxygen peaks from cotton. The nitrogen was from the amide group of PNIPAAm. The presence of Ag proves that the existence of Ag on the cotton surface.

#### 3.6.4. Determination of the Grafting Rate of the IPN Hydrogel Modified Cotton

From Table 2, it can be seen that the grafting rate of the cotton fabric was increased by increasing the amount of SS in the IPN hydrogel. The grafting rate of the cotton fabric modified by the PNIPAAm hydrogel was poor, due to the lack of active groups in the PNIPAAm hydrogel for cotton fabrics. However, the SS in the IPN hydrogel contained a large number of –NH_2_, –COOH, and –OH groups. In addition, the cotton fiber contained a lot of –OH groups. Therefore, under the action of a cross-linking agent, the IPN hydrogel can be grafted onto the cotton, as Figure 11 shows. The grafting rate did not change significantly, even after the IPN hydrogel grafted cotton fabric was cleaned five times using distilled water at room temperature, indicating strong covalent bonding. 

#### 3.6.5. The Contact Angle of IPN Hydrogel Modified Cotton

**Figure 13:**
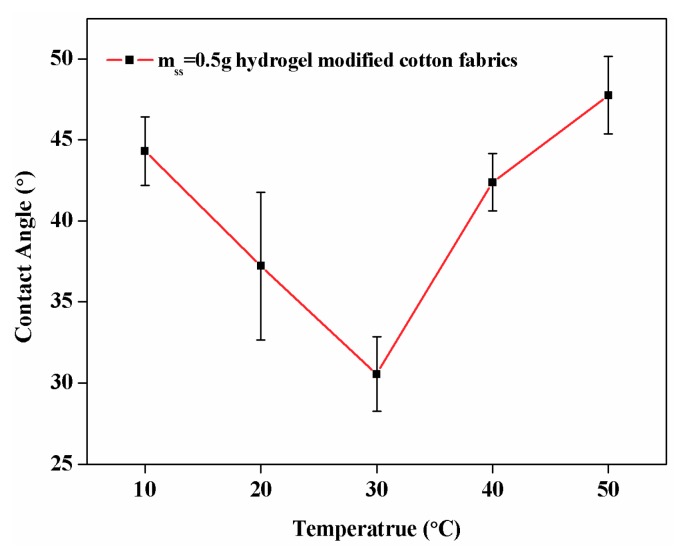
The Contact Angle of IPN hydrogel modified cotton at different temperature.

The temperature-sensitive behavior of the cotton modified by IPN hydrogel was characterized by the contact angle test method for hydrophilicity and hydrophobicity at different temperatures (Figure 13). Because both SS and cotton fabrics contained a large amount of hydroxyl groups, they exhibited hydrophilicity at each temperature. However, their hydrophilicities were different. At the temperature close to LCST (≈31 °C), the entire system was in a stretched state, and easily absorbed water molecules. However, the hydrophilicity tended to decrease when the temperature was higher or lower than the LCST because the external temperature stimulated the contraction of molecular chains. Therefore, it can be confirmed from the contact angle that a cotton fabric having temperature sensitivity was synthesized.

#### 3.6.6. Antibacterial Test of IPN Hydrogel Modified Cotton

As listed in Table 3, the antibacterial rate of the m_ss_ = 0.5 g hydrogel modified cotton against *E. coli* and *S. aureus* was more than 10%. The antibacterial rate of the m_ss_ = 0.5 g/Ag hydrogel modified cotton against *E. coli* and *S. aureus* was as high as 99%, which is mainly attributed to the following two aspects:

(1) The reducing effect of SS was a decisive factor in the antibacterial effect of silver. AgNPs had high specific surface area in contact with bacteria due to its small particle size. Ag has a high catalytic ability and oxygen atoms have strong oxidizing properties. Thus, the sterilization effect is achieved. 

(2) When silver ions were released from the dead bacteria, they came into contact with other bacteria again. This behavior also reflects the durability of inorganic antimicrobial agents.

In conclusion, the amount of sericin used in the experiment did not acquire the antibacterial effect of cotton fabrics; the main antibacterial effect came from nano silver.

## 4. Conclusions

In this study, an IPN hydrogel was prepared by combining PNIPAAm with SS. The LCST of the IPN hydrogel was 31 °C. The two components of SS and PNIPAAm had good compatibility with each other. The T_g_ of the IPN hydrogel increased by increasing SS content. Using SS as the reducing agent and dispersant, Ag^+^ was reduced to Ag^0^ by in-situ reduction. Then, PNIPAAm and SS/Ag^0^ were combined by interpenetrating network technology to prepare the IPN hydrogel having an antibacterial agent. The IPN hydrogel was successfully grafted onto the cotton surface by cross-linking reaction. The IPN hydrogel integrated well with the cotton without damaging the fabric’s structure. Due to the presence of silver ions, the modified cotton had a good antibacterial effect. The cotton was modified by an IPN hydrogel to have both temperature-sensitive and antibacterial properties.

This study is the beginning of the fabrication of smart textiles consisting of fabric coated with temperature-responsive IPN hydrogels. However, comprehensive testing of modified cotton fabric would be required prior to their use as medical dressings. Other applications of IPN hydrogel-modified cotton fabrics such as swelling behavior, mechanical properties, biocompatibility, and advanced comfort capabilities, will be reported in subsequent manuscripts.

## Figures and Tables

**Table 1:** The elements content on IPN hydrogel-modified cotton from EDS.

Simple	Surface Element Content/%			
unmodified cotton	C:54.33	O:45.67	N:0	Ag:0
IPN hydrogel-modified cotton	C:48.28	O:42.16	N:9.32	Ag:0.24

**Table 2:** Grafting degree of m_ss_ = 0.5 g hydrogel modified cotton.

Modified Cotton	D_G_/% (Before Washing)	D_G_/% (After Five Times Washing)
m_ss_ = 0 g	0.75 ± 0.1	0.1 ± 0.1
m_ss_ = 0.3 g	13.17 ± 0.4	11.65 ± 0.6
m_ss_ = 0.4 g	13.70 ± 0.3	12.03 ± 0.5
m_ss_ = 0.5 g	16.45 ± 0.5	14.21 ± 0.6
m_ss_ = 0.6 g	20.22 ± 0.3	17.95 ± 0.4

Note: Unwashed refers to direct weighing without treatment after treatment.

**Table 3:** Bacterial reductions of the IPN hydrogel modified cotton.

*S. aureus*		*E. coli*	
Unmodified cotton (cfu/mL)	1.47 × 10^6^	Unmodified cotton (cfu/mL)	1.28 × 10^6^
m_ss_ = 0.5 g hydrogel modified cotton (cfu/mL)	1.56 × 10^5^	m_ss_ = 0.5 g hydrogel modified cotton (cfu/mL)	1.43 × 10^5^
Bacterial reduction (%)	10.61	Bacterial reduction (%)	11.17
m_ss_ = 0.5 g/Ag hydrogel modified cotton (cfu/mL)	2.67 × 10^3^	m_ss_ = 0.5 g/Ag hydrogel modifed cotton (cfu/mL)	2.71 × 10^3^
Bacterial reduction (%)	99.81	Bacterial reduction (%)	99.79
